# New York Society of German Physicians

**Published:** 1880-12

**Authors:** 


### NEW YORK SOCIETY OF GERMAN PHYSICIANS.

*Multiple Uterine Fibroids, with Calcareous Deposits.*—Dr. Tauszky presented the uterus of a colored woman, showing numerous interstitial fibroids in a condition of calcareous change.

Dr. Tauszky exhibited a second specimen, which consisted of a membranous cast of the uterine cavity of a young lady nineteen years of age. The lady in question was said to suffer from membranous dysmenorrhœa, and it was stated that a similar membrane was shed at each monthly epoch. The treatment of this affection was also alluded to.

*Removal of Steel Splinter from Vitreous Humor.*—Dr. Oppenheimer exhibited a patient from whose eye a steel splinter had been extracted with Gruening's magnet.

*Large Uterine Fibroid.*—Dr. Scharlan presented a large uterine fibroid which he had lately successfully removed. He stated that the presence of the tumor had been recognized sixteen years ago by Dr. Kimmerer.

Dr. Kucher demonstrated the mode of application and action of Poulet's obstetric forceps.

*Sudden Amaurosis Following the Administration of Quinine.*—Dr. Gruening related this case, and stated that, in his opinion, the amaurosis was unquestionably the direct result of the action of quinine. The following history was given: Mrs ———. aged thirty-five years, was obliged to have an abortion performed on her. Five days after this operation, the uterine cavity was scraped with a curette on account of the continuance of an ichorous discharge. This procedure was followed by slight hemorrhage. The temperature now rising to 106° F., the local treatment was supplemented by the administration, in thirty hours, of eighty grains of quinine. After severe hysterical seizure the patient now suddenly became deaf and blind. In the course of twenty-four hours the patient's hearing returned. The eyes were at that time in the following condition: pupils dilated to their fullest extent, absolutely without reaction. The different media of the eye were normally transparent, but the papilla showed whitish discoloration, and the retinal vessels were considerably attenuated. The external portion of the retina was somewhat opaque, the macula distinctly red and projecting; absence of corneal anæsthesia. A few days later the patient stated that her vision returned for some time, but this was not proven to be the case. After three weeks, however, she was again able to see, but once more became blind with the reappearance of her menstruation. These occurrences were again repeated at a subsequent menstrual epoch. The patient then began to improve slowly, and, under the administration of nitrite of amyl, digitalis, iron, strychnia, and galvanism, fairly progressed to recovery.

In his further remarks upon such rare instances of quinine amaurosis, Dr. Gruening said that the prognosis of these cases was not unfavorable. The literature of the subject embraced five cases with partial recovery, *i. e.*, in all the field of vision remained permanently diminished. In the present instance, while central vision was perfect, the field of vision was narrowed, and in addition the patient was color-blind. The pupils also showed absence of dilatation after the use of eserin, but the normal calibre did not return with the cessation of the drug; they remained visibly enlarged. Light produced no effect upon their size, but during convergent motions reaction occurred. Finally, he said that the permanence of the diminution of the visual field was so characteristic of quinine amaurosis, that this alone would suffice to exclude sepsis, hysteria, and anæmia from the list of possible etiological factors in the present case.

*Diphtheritic Exanthemata.*—Dr. Jacobi related the history of a case recently observed by him, which he was inclined to regard as an instance of diphtheria accompanied by a peculiar rash. He stated that in former years such cases had more frequently come under his notice. To distinguish this affection from scarlatina with diphtheria, it must be remembered that the former was characterized by a rash lacking the uniformity of scarlatinous exanthemata, that it made its first appearance on the face and feet of the patient, that it frequently assumed the appearance of an urticaria sprinkled over with pustules of fluid contents. Moreover, the diphtheritic exanthem was not accompanied by the well-known appearances of the mucous membrane of the mouth and tongue. He also emphasized the additional point that diphtheria rarely made its appearance on the first day of an attack of scarlet fever. Diphtheritic exanthemata usually occurred in the early stages of the disease, commonly in cases remarkable for the absence of all septic symptoms. The hemorrhagic rashes of the later stages were of serious, often fatal significance. Thus Callimani reported 115 deaths in an epidemic of 200 cases.

Dr. Jacobi observed that the case of diphtheritic exanthem reported by him at the last meeting, subsequently turned out to be an ordinary case of scarlet fever, showing that strikingly characteristic symptoms may mislead one to form an incorrect judgment.

*Perforation of Vermiform Appendix.*—Dr. Gleuck presented a specimen of the above, removed from the body of a boy aged seven years, who had recently fallen a victim to perityphlitis with perforation. The attack had been a very acute one, and had proved rapidly fatal. The appendix showed two perforations, and contained hardened fecal matter, embedded in which there were found poppy and anise seeds and grape-pits It was thought probable that a slow ulceration without apparent symptoms had preceded the sudden perforation. The immediate cause of the latter was supposed to be a jumping of the boy from the back of his father.

Dr. Jacobi remarked that in children between three and eleven years of age, it was a normal occurrence to find a fold of mucous membrane at the entrance of the appendix, and that this fold, acting as a valve, would cause retention in the appendix of such particles as might accidentally have entered it. Moreover, it should be born in mind that peritonitic adhesions frequently brought about permanent flexions of the appendix. The late Dr. Krakowitzer had expressed an opinion to this effect—that, as a rule, perforation of the vermiform appendix was produced by local peritonitis.

Dr. Kucher stated that congenital flexions of this organ had also been observed.

Dr. Jacobi said that in future he would practice abdominal section and drainage, as soon as the diagnosis was fairly established in such cases In answer to a question from Dr. Mantner, he added that he should not consider it necessary to ligate the appendix, because adhesions would be likely to close the perforated points as soon as antiseptic treatment was begun.

*Double Invagination of Small Intestine, with Ulcerated Mucous Polypus.*—Dr. Wendt presented a specimen of the above, which had been previously exhibited at a meeting of the Pathological Society.

Dr. Jacobi thought that the ulceration caused by the intestinal polypus had produced a local paralysis of the muscular coat of the intestine, and that this had led to the invagination of the gut.

*Large Semi-Solid Ovarian Tumor.*—Dr. Wendt presented a specimen, consisting of a portion of an enormous ovarian tumor, successfully removed by Dr. Bopp, of the German Hospital. The history of the case, condensed from the hospital records, was as follows: Mrs. ———, aged thirty-eight years, has given birth to ten children. Menstruation, pregnancies, labor, confinement, were always normal. Last January she had an attack of peritonitis, but remembered no other serious disease. A tumor was first noticed after her last confinement, and from that time on her abdomen grew steadily larger.

On her admission to the hospital the abdomen was found to be enormously distended, and through its walls a fluctuating tumor was palpated. The surface was not even, and the consistence did not appear to be the same at all points. No fluid escaped after the introduction of a good-sized trocar.

On the 19th of April ovariotomy was performed in the ordinary manner, and under strictly antiseptic precautions. When the cyst was exposed to view, a large Spencer Well's trocar was introduced, but no contents flowed out. The tumor had to be incised and part of its gelatinous and sticky contents removed by using the hand as a scoop. The tumor was found to weigh twenty-eight pounds. The broad pedicle was dropped into the pelvic cavity, and the wound closed by silver wire and catgut sutures. The progress of the case after the operation was favorable. Only on one day the temperature rose to 104° F. The patient was discharged cured in June. The examination of the tumor showed it to consist of many rounded partitions of various sizes, filled with a semi-solid, whitish, glistening mass, having in many places the appearance and consistence of white cerebral substance. Microscopically this mass was composed of variously-shaped epithelial cells, the majority of which showed colloid degeneration. Many of these bodies were also found in different stages of fatty degeneration. Portions of the tumor, when hardened in alcohol, shrunk to about one-tenth their original size, and microscopic sections then exhibited the following appearances: From the external fibrous capsule, ramifying trabecules of connective tissue proceeded inwards, growing, thinner as the central portions of a partition were approached. These stalks of connective tissue had a single or double lining of long, slender, cylindrical epithelia, containing large, rounded or oval nulcei. Their protoplasm appeared homogeneous or faintly granular. Beyond this layer of epithelia a more or less homogeneous mass was found filling out the interspaces of neighboring trabeculæ. Here and there in this mass of delicate outlines of cells could still be discovered, showing that its origin was undoubtedly colloid metamorphosis of the lining epithelia. Many trabecules were quite vascular, and in some minute hemorrhages had taken place.

Tumors of this kind had been observed by Ohlshausen and others, but, when of this large size, they had invariably proceeded from the parovaries, and were generally bilateral. In this case the tumor was of true ovarian origin, and this fact, together with its large size, constituted its exceptional nature. Another feature of interest was, that although the tumor contained no fluid, it had given unmistakable signs of fluctuation. Such tumors were called by German observers *cystoma papillare proliferum*, or simply *papilloma proliferum*. They were really proliferous adenomata with secondary colloid degeneration.

Dr. Caille observed that he had met with similar microscopic appearances in the early stage of a cystic goitre.

Dr. Mantner called attention to the fact that Ohlshausen had inferred the parovarian nature of such rare tumors from the presence of ciliated epithelia, and asked whether this had been found in the present instance.

Dr. Wendt answered that a careful examination had failed to reveal their presence, and that there was not the least doubt about the ovarian origin of the tumor.

*Model of Spinal Cord.*—Dr. Seesel demonstrated an ingenious model, showing the course and direction of the various columns and fibres of the spinal cord.

Dr. Mandelbaum referred to three tracheotomies which he recently had occasion to perform. The first operation was upon a deaf-mute. Two weeks after the tracheotomy singultus occurred, which could be relieved by closure of the canula. In the fourth week the tracheotomy tube became clogged up with granulations. Speedy cure took place upon the removal of these. The second case was somewhat similar and likewise recovered In the third case death took place.—*N. Y. Medical Record.*

